# Anatomically Accurate, High-Resolution Modeling of the Human Index Finger Using In Vivo Magnetic Resonance Imaging

**DOI:** 10.3390/tomography8050196

**Published:** 2022-09-21

**Authors:** Luka Rogelj, Rok Dolenec, Martina Vivoda Tomšič, Elmar Laistler, Urban Simončič, Matija Milanič, Rok Hren

**Affiliations:** 1 Faculty of Mathematics and Physics, University of Ljubljana, 1000 Ljubljana, Slovenia; 2 Jozef Stefan Institute, 1000 Ljubljana, Slovenia; 3 University Clinic of Respiratory and Allergic Diseases Golnik, 4204 Golnik, Slovenia; 4 Faculty of Medicine, University of Ljubljana, Vrazov trg 2, 1000 Ljubljana, Slovenia; 5 High Field MR Center, Center for Medical Physics and Biomedical Engineering, Medical University of Vienna, 1090 Vienna, Austria; 6 Institute of Mathematics, Physics, and Mechanics, 1000 Ljubljana, Slovenia

**Keywords:** segmentation, high-resolution MRI, simulation

## Abstract

Anatomically accurate models of a human finger can be useful in simulating various disorders. In order to have potential clinical value, such models need to include a large number of tissue types, identified by an experienced professional, and should be versatile enough to be readily tailored to specific pathologies. Magnetic resonance images were acquired at ultrahigh magnetic field (7 T) with a radio-frequency coil specially designed for finger imaging. Segmentation was carried out under the supervision of an experienced radiologist to accurately capture various tissue types (TTs). The final segmented model of the human index finger had a spatial resolution of 0.2 mm and included 6,809,600 voxels. In total, 15 TTs were identified: subcutis, Pacinian corpuscle, nerve, vein, artery, tendon, collateral ligament, volar plate, pulley A4, bone, cartilage, synovial cavity, joint capsule, epidermis and dermis. The model was applied to the conditions of arthritic joint, ruptured tendon and variations in the geometry of a finger. High-resolution magnetic resonance images along with careful segmentation proved useful in the construction of an anatomically accurate model of the human index finger. An example illustrating the utility of the model in biomedical applications is shown. As the model includes a number of tissue types, it may present a solid foundation for future simulations of various musculoskeletal disease processes in human joints.

## 1. Introduction

Early detection of bone erosion and inflammation in finger joints due to a number of pathologies [1,2,3] is one of the critical factors for timely treatment and improved long-term functional outcomes. Magnetic resonance (MR) imaging may be used for detecting such abnormalities; however, until recently, relatively poor resolution presented an obstacle to its clinical use when applied to finger joints, especially in 1.5 T MR units, which are commonly installed in hospitals [4,5]. MR images can be used to segment various body parts such as the brain [6], liver [7], lung [8] and torso [9].

Laistler and coworkers [10] used a specially designed coil geometry parametrization in order to obtain high-resolution, in vivo MR images of a finger. In image post-processing, they constructed a model of the finger with eight different tissue types (TTs). In this study, we are building upon their work with the goal of constructing a comprehensive and anatomically accurate, high-resolution model of the human finger. The primary objective of our study was to maximize the number of tissue types in the model while performing the segmentation under the supervision of an experienced radiologist, as an anatomically accurate model is a prerequisite for various simulation studies. The secondary objective was to test the versatility of the model, particularly with regard to, inter alia, simulation of inflammation of finger joints.

## 2. Materials and Methods

### 2.1. Data

The basis of our segmented model was the data collected by Laistler et al. [10]. MR images were captured at ultrahigh magnetic field (7 T) with a radio-frequency coil specially designed for finger imaging. For better homogeneity of the field in the coil, the windings were denser at the edges and sparser at the center, optimized by quasi-static simulations using the Biot–Savart law.

The index finger inserted in the coil was covered by a 5 mm MR-invisible foam padding, with the purpose of better stabilizing the finger. The coil was then inserted in a 7 T MRI system (Magnetom 7 T, Siemens Healthineers, Erlangen, Germany). The imaging protocol used for high-resolution imaging of the human finger was a three-dimensional magnetization-prepared rapid gradient echo (MP-RAGE) sequence with fat suppression, a repetition time (TR) of 2730 ms, inversion time (TI) of 1700 ms, echo time (TE) of 6.71 ms and acquisition time (TA) of 8 min 45 s. The field of view (FOV) of the final image was 100 mm × 50 mm with a resolution of 195 μm × 195 μm × 200 μm, which was deemed high enough to perform a detailed segmentation [11].

### 2.2. Segmentation

We carried out segmentation using a free, open-source software system for interactive development of medical images (Medical Imaging Toolkit MITK 2016.3.0, Heidelberg, Germany) [12]. In order to accurately capture various TTs, the overall process of segmentation was supervised by a radiologist with 8 years of experience in musculoskeletal imaging.

The segmentation was performed using the MITK tool 3D Region Growing or 2D Region Growing [13], which approximated—based on the gray-value intervals—the TT regions; segmentation was then finalized by the radiologist. Such an approach was used for the following TTs: skin, cartilage, bone, vessels, tendons and Pacinian corpuscle. For those TTs with low contrast in MR images (nerve, collateral ligament, volar plate, pulley A4, synovial cavity and synovial membrane), segmentation had to be done manually by a radiologist, who employed the mouse cursor. Special care was taken in the segmentation of finger joints.

**Figure 1:**
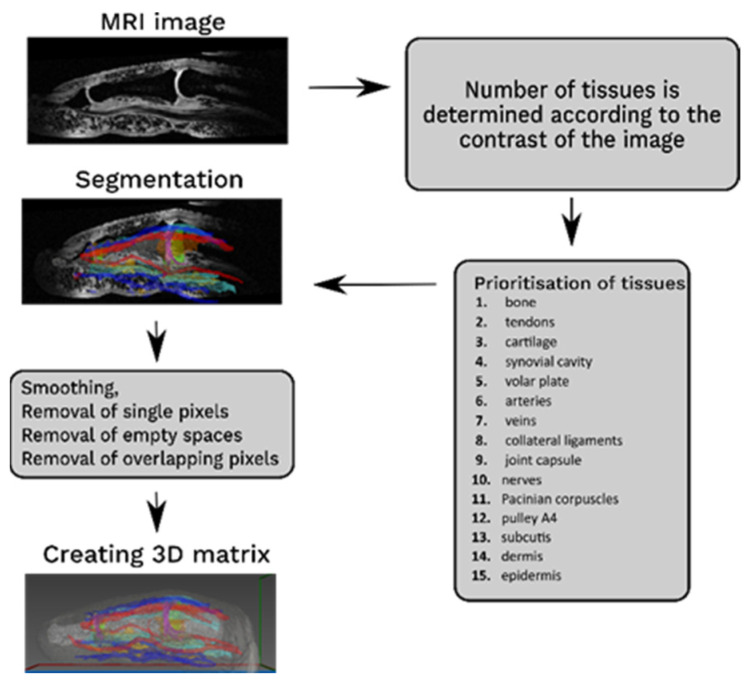
Segmentation protocol, in which segmented tissues were prioritized, smoothed, and transformed into a 3D matrix.

The segmentation was done in a slice-by-slice manner [14] following the protocol presented in Figure 1. The number of tissue types and the segmentation sequence were determined by the radiologist, with tissues having the highest contrast in MR images being segmented first. The segmented tissues were further smoothed; single pixels were removed, and unnecessary missing pixels were accounted for. Individual segments were imported to MATLAB (MathWorks, Natick, MA, USA), in which overlapping pixels were assigned according to a tissue prioritization list; finally, a 3D matrix was created in which each tissue was represented by a single value from 1 to 15.

The quality of the segmentation was defined by the details, which were segmented, and to that end, the supervision of an experienced radiologist was imperative. It is important to note that some TTs (e.g., tendons, cartilage, vessels) had in some regions a thickness of only one or two voxels.

For purposes of visualization of the model, we used an MITK tool, based on the marching cube algorithm that created a polygonal mesh from voxel data [15]. The surface was then exported as a stereolithography (.STL) file to ParaView (https://www.paraview.org/ accessed on 2 May 2022) where a Laplacian smoothing filter was applied, and individual colors were assigned to each TT.

### 2.3. Versatility of the Model

To test the versatility of the model of an index finger, we tailored it to the conditions of arthritic joint, ruptured tendon, and variations in the geometry.

### 2.4. Application of the Model

To show the utility of the model, we applied a custom-weighted photon 3D Monte Carlo simulation of optical transport through the human index finger. The custom-made software was based on the Monte Carlo model of steady-state light transport in multilayered tissue (MCML) [16], a well-established and validated code in the field of biomedical optics, which we extended for computations in fully 3D voxelated geometry and for parallel execution via several processor cores of a graphics card. Details of the custom simulation are presented in our previous work [17]. Briefly, the algorithm assumed homogeneity of optical properties of the tissue within each voxel of the model. Packets of photons with a weight of one were generated in the form of a perfectly collimated light source uniformly distributed over the whole geometry and transported through the simulated model. According to the simulated optical properties (absorption and scattering coefficients, refractive index and anisotropy), the photons were reflected, refracted or transported at voxel boundaries, and a fraction of the packet’s weight was absorbed in each voxel. Photon packets were stopped when their weight fell below a certain threshold. A mirror boundary condition was applied whenever the model geometry extended beyond the limits of the simulated volume. Transmittance and reflectance images were formed by adding the weights of packets exiting the model in the forward or backward direction, respectively.

The models of arthritic disease in a human finger were constructed by applying linear scaling transformations on transverse slices of the base finger geometry. The shape and magnitude of these transformations were determined by considering the expected morphological changes in arthritic joints, presented in more detail in our previous work [18]. The change to the base geometry consisted of swelling of the synovial fluid (volume increased by 7.5 times) and synovial membrane (3 times), with the other tissues displaced accordingly. The optical properties of tissues were simulated as precisely as possible from the available experimental data, while the expected changes due to arthritic disease were also included [18]. The optical properties for a healthy finger are presented in Appendix A.

The simulation code was developed in C++ and compiled on a Linux operating system using a CUDA toolkit v8.0 (NVidia, Santa Clara, CA, USA), with the CUDA compute capability set to version 3.0. The transmittance and reflectance images were obtained by running simulations in the 400 nm to 1100 nm spectral range, with 10 nm steps; at each wavelength, 10^8^ photons were tracked. Simulations were run on an NVidia GeForce GTX TITAN X graphics card with 3072 computation cores enabling massive simulation speedup compared to the original MCML program.

In order to illustrate the utility of our model, we compared the models of healthy and inflamed human fingers to results obtained by a custom-made laboratory hyperspectral imaging (HSI) system [19] in a subject with confirmed arthritis diagnosis; the subject signed an informed consent form. The HSI images were obtained between 400 nm and 1000 nm with a 0.3 nm step, with an integration time of 300 ms per line. To image the area of the finger joint, 500 lines were acquired, with step size matching the system’s spatial resolution of 0.065 mm; including time required to scan the imaging head, this resulted in total imaging time of 5 min per finger.

## 3. Results

### 3.1. Healthy Finger

The final segmented model of the human index finger had a spatial resolution of 0.2 mm and included 6,809,600 voxels. The following 15 TTs were identified: subcutis, Pacinian corpuscle, nerve, vein, artery, tendon, collateral ligament, volar plate, pulley A4, bone, cartilage, synovial cavity, synovial membrane, epidermis and dermis.

**Figure 2:**
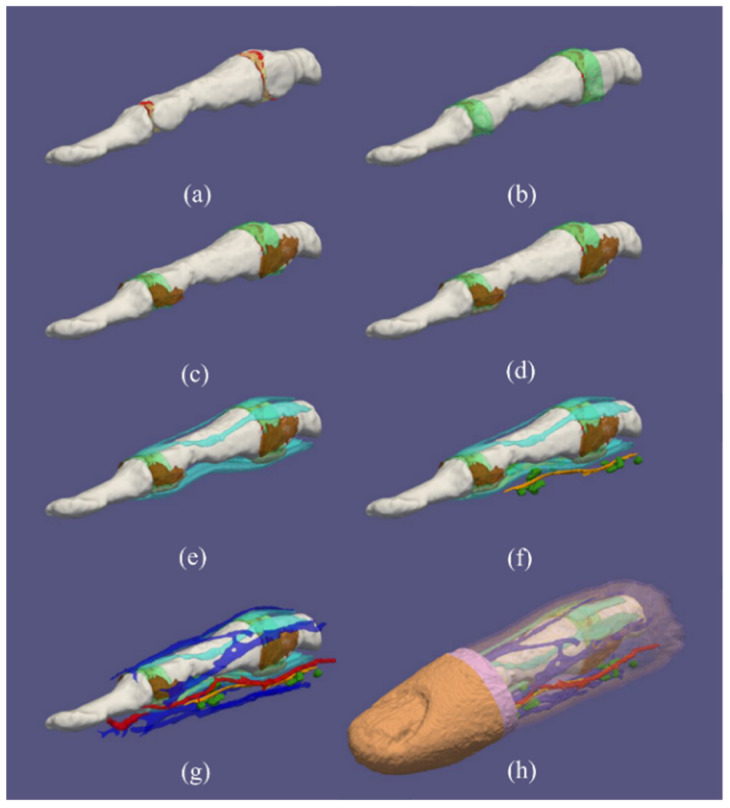
Three-dimensional rendering of the human index finger model from the middle part of the proximal phalanx to the distal phalanx depicting different tissue types (TTs): (**a**) cartilage (red), synovial cavity (yellow), (**b**) joint capsule (light green), (**c**) collateral ligaments (brown), (**d**) volar plate (beige), (**e**) tendons (turquoise), (**f**) nerves (orange) and Pacinian corpuscles (dark green), (**g**) arteries (red) and veins (dark blue), (**h**) epidermis (orange) and dermis (pink) with all other TTs including subcutis (mauve).

**Figure 3:**
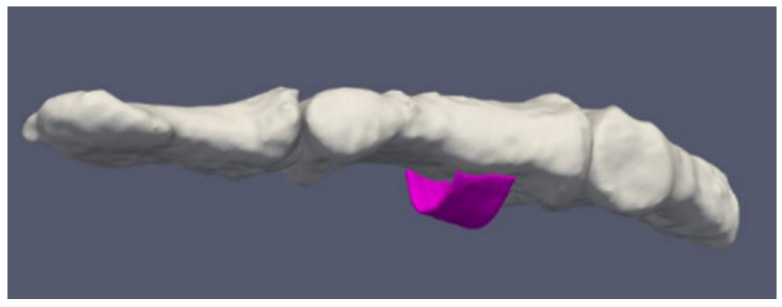
Three-dimensional rendering of the human index finger model showing the bone structure only and pulley A4.

Figure 2 shows a three-dimensional rendering of the segmented model separately for different TTs, starting with finger-joint TTs (cartilage with synovial cavity) (Figure 2a) through finger-surface TTs (dermis, epidermis) (Figure 2h). We made every attempt to incorporate all the relevant TTs in the model, which were visible on the high-resolution MR images. One of the advantages is that we modeled the finger joints by including cartilage, synovial cavities, joint capsules, collateral ligaments and volar plates. As depicted in Figure 3, we were even able to reconstruct highly detailed anatomical structures, such as pulley A4. Overall, our model closely corresponds to the textbook anatomy.

### 3.2. Arthritic Finger Joints

**Figure 4:**
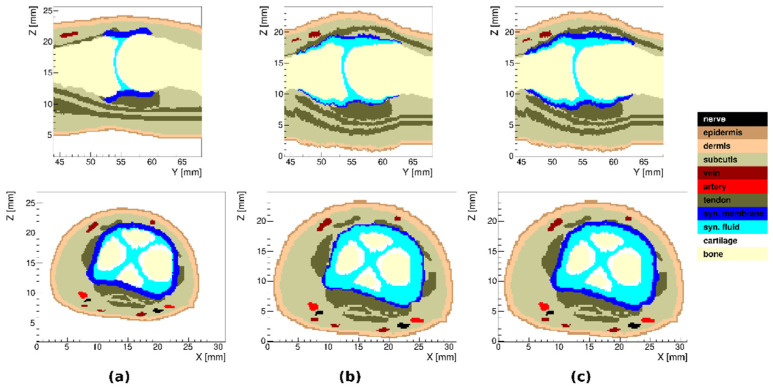
Sagittal and transverse cross sections of the proximal interphalangeal joint region in the model of arthritic index finger: (**a**) with synovial membrane thickening, (**b**) with synovial fluid effusion and (**c**) with both synovial membrane thickening and synovial fluid effusion.

In the model of arthritic finger joints, we used a subset of 11 TTs (combining tendon, collateral ligament, volar plate and pulley A4 and combining Pacinian corpuscle and nerve) to achieve clearer visualization. Three different models of joint inflammation were created: (i) with synovial membrane thickening, (ii) with synovial fluid effusion and (iii) with both synovial membrane thickening and synovial fluid effusion. In all three cases, only inflammation in the proximal interphalangeal joint was considered. The resulting models are presented in Figure 4.

### 3.3. Simulated Ruptured Finger Tendon

**Figure 5:**
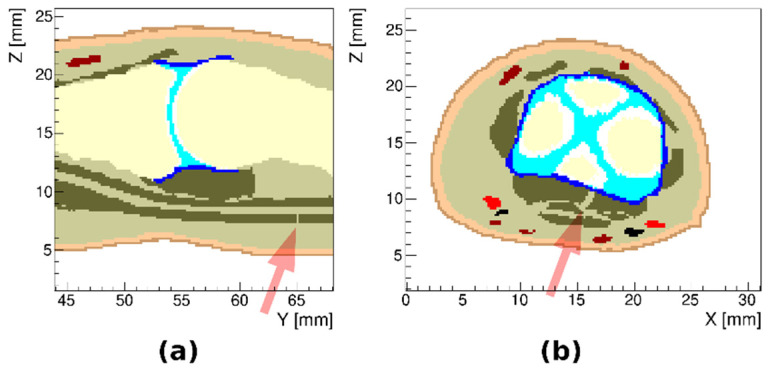
Two models of ruptured tendons in the index finger, first shown in transverse (**a**) and the second in sagittal (**b**) cross sections of the proximal interphalangeal joint region. The sites of ruptures are shown with an arrow.

Figure 5 shows examples of a simulated ruptured finger tendon. We manipulated individual voxels to represent breaks in selected tendons and did not take into account tendon contraction or deformation after the rupture.

### 3.4. Variations in the Geometry of a Finger

**Figure 6:**
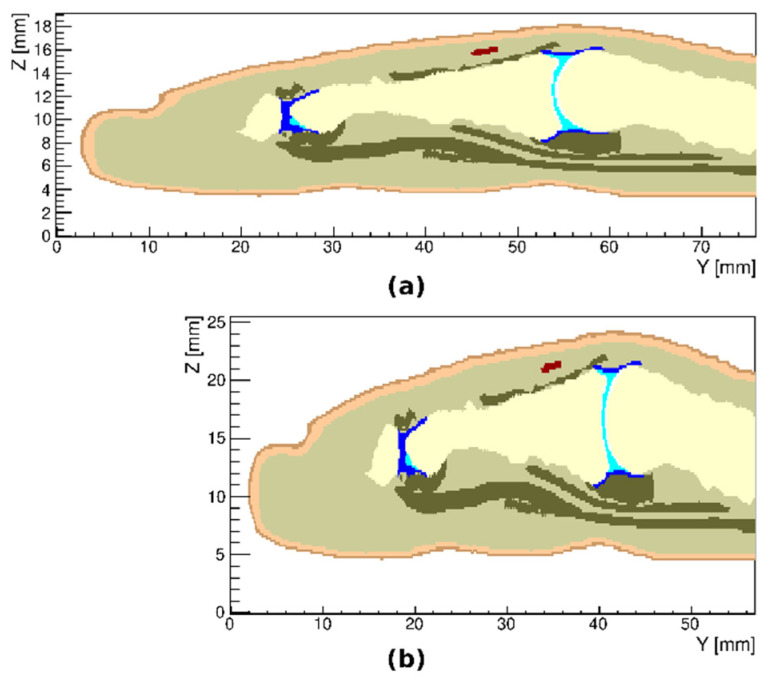
Sagittal cross sections of the model of an index finger, illustrating its tailoring to variations in geometry: scaled by 75% in the z-direction (**a**) and scaled by 75% in the y-direction (**b**).

As our model allowed the changing of voxel dimensions, it was rather easy to make an adjustment due to variations in the geometry of an index finger; examples are shown in Figure 6.

### 3.5. Monte Carlo Simulations of Optical Transmission

**Figure 7:**
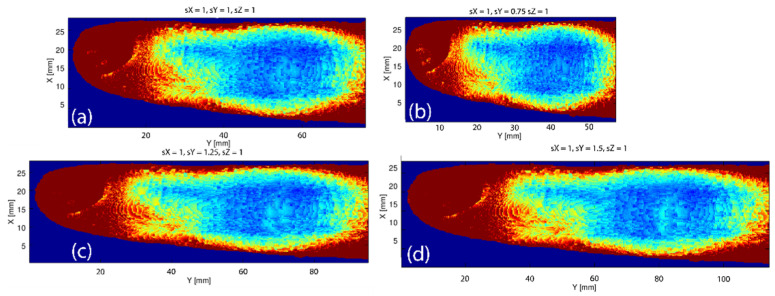
Results of Monte Carlo simulations of optical transmission through the model of human index finger at 860 nm with geometry scaled along the long axis of the finger (in the y-direction): (**a**) base case; (**b**) scaling of the model by 75% along the *y* axis; (**c**) scaling of the model by 125% along the *y* axis; (**d**) scaling of the model by 150% along the *y* axis.

Figure 7 illustrates the results of Monte Carlo simulations of optical transmission through our model, in which geometry was scaled along the long axis of the finger. We can clearly see that transmittance is larger near the edges of the finger than in its central parts, and the shorter the finger, the more pronounced is the reduction of the transmittance. A bright spot can be observed on the joint location, which is a consequence of light passing through the synovial cavity.

**Figure 8:**
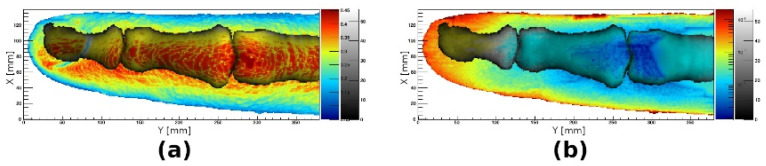
Reflectance (**a**) and transmittance (**b**) images resulting from optical simulation using the index finger model. Images are overlaid in grayscale showing the total thickness of the bone tissue in the model.

The human index finger model was used to study the effects that the geometry and optical properties of different parts of anatomy have on optical reflectance and transmittance of the whole finger. Figure 8 shows an example of such images when the effect of the bones was studied. As described previously, the properties or geometry of different tissues of the model can be arbitrarily adjusted to reflect a wide variety of injuries, illnesses or simply population variability. The finger model can, thus, be used to exactly correlate possible observables with such changes.

### 3.6. Tissue Distribution

**Figure 9:**
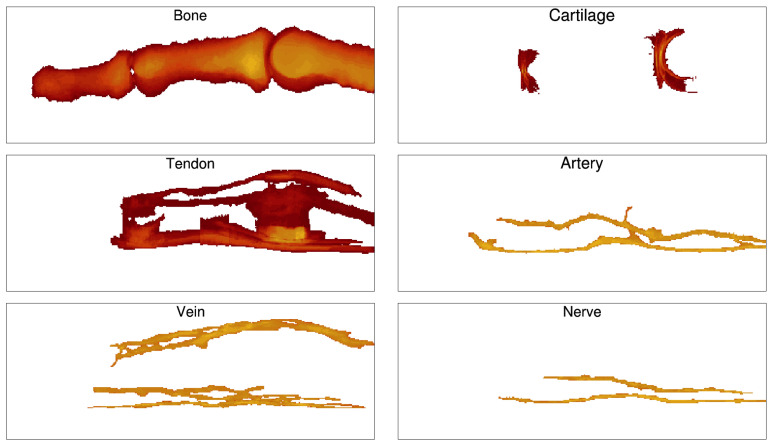
Sagittal projections showing total thickness of different tissues in the index finger model.

Several applications may require information on the distribution of a specific tissue in the finger, for example, calculated in different projections for tomography reconstruction. The human index finger model can easily provide such information, for all segmented tissues. In Figure 9, sagittal projections of total thickness are shown for six different tissues of the model.

### 3.7. Comparison of Simulations with Hyperspectral Imaging of Healthy and Arthritic Fingers

**Figure 10:**
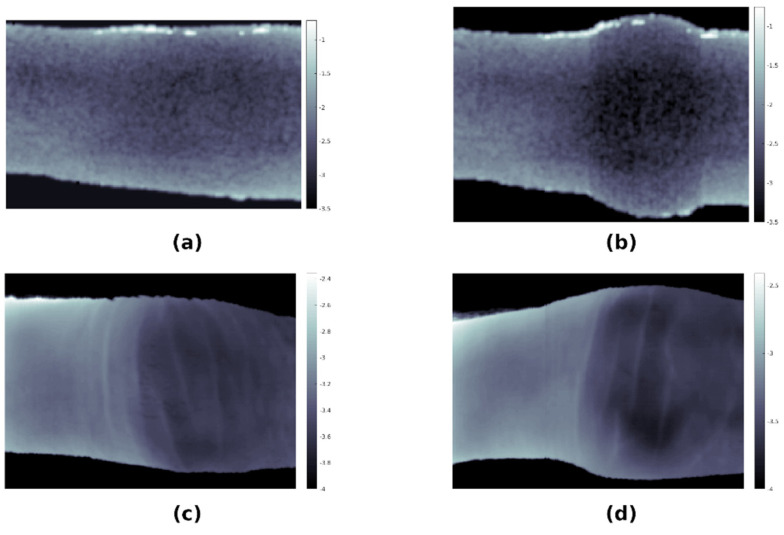
Transmittance images (logarithmic scale) obtained using simulations (**a**,**b**) and custom-made laboratory hyperspectral imaging (HSI) system (**c**,**d**) for a healthy finger (**a**,**c**) and a finger affected by rheumatoid arthritis (**b**,**d**). All images include data integrated for wavelengths between 650 nm and 760 nm, a spectral range found to be most sensitive to changes due to arthritic disease.

To test the merit of the model, a custom-made laboratory HSI system was applied to image a healthy and an inflamed finger in a human subject with a confirmed arthritis diagnosis and compared to simulations. At the time of imaging, each individual finger of the subject was graded by a medical doctor on the EULAR-OMERACT scoring system. Figure 10 shows a comparison of experimentally obtained transmittance images and simulated images for healthy (EULAR-OMERACT score of 0) and affected (EULAR-OMERACT score of 2) fingers of the same patient. As the model had a lower resolution than the HSI system (0.2 mm vs. 0.065 mm), the very fine structure of the skin surface visible in experimental images was not reproduced by the simulations; however, the swelling of the finger and increase in optical absorption due to inflammation was consistent between simulations and experimental data.

## 4. Discussion

To our knowledge, this is the first report of an anatomically accurate, high-resolution model of a human index finger. As shown elsewhere, anatomically detailed models of joints may be highly useful in simulating musculoskeletal disease processes [20,21]. Until recently, such studies were precluded from being applied to finger joints due to the insufficient spatial resolution of MR imaging.

In high-field MR imaging, the influence of local variations in magnetic susceptibility can play a decisive role in imaging, including the lack of possibility of quantitative comparisons [22]. The artifacts arising from magnetic susceptibility differences between different tissue types generally increase with magnetic field strength and, depending on the acquisition sequence, can result in image distortion and local signal loss. Acquisition used in this research is not particularly prone to susceptibility artifacts due to its high resolution and low echo time.

The spatial resolution of the MR images underlying this study is 195 × 195 × 200 µm^3^, and the segmentation of a large variety of structures was possible, from the fingertip to approximately the center of the proximal phalanx. Further improvement of the spatial resolution such as in [23] might facilitate the segmentation of finer structures. Currently, the limitations in spatial resolution for in vivo finger MR imaging are subject motion, RF coil sensitivity and gradient strength. These could be overcome by restricting subject motion by a more effective finger fixation system or even applying post-mortem imaging, developing a dedicated, phased-array finger coil and more powerful gradient systems. However, such surface coil arrays tailored to the human finger are not yet readily available and could lead to less-uniform image homogeneity, likely complicating image segmentation. Unfortunately, dedicated high-performance gradient systems for the finger are probably out of reach given the rather small community for finger imaging.

The field of view of the coil used in this study has limitations in length along the finger, as seen from the lower signal intensity toward the distal and proximal ends of the finger. This could also be overcome by a newly designed coil with a larger field of view.

An attractive area for expanding the model includes using diffusion tensor imaging (DTI), which would enable, e.g., simulations of nerve signals. DTI brings its own challenges due to large magnetic field gradients [24,25], which necessitate the estimation and elimination of systematic errors [26]. DTI alone could also provide valuable information regarding the anisotropy of tissue physical properties (e.g., thermal conductivity, elastic properties), which could then be built into an advanced anisotropic model of the human index finger. However, these steps are not trivial and would require much additional research work.

When comparing our segmentation model with that of Laistler et al. [10], the following main differences can be ascertained: our model uses 15 TTs versus 8 TTs and explicitly incorporates finger joint structures, such as the synovial cavity and synovial membrane. As shown here, this has proven to be particularly important when simulating the inflammation process in the finger joints observed in rheumatoid arthritis.

There have been several studies using MR images for the segmentation of complex body parts, e.g., the shoulder [27] and knee [28]. These papers used images of lower resolution with segmentation limited to the most prominent TTs, thereby neglecting fine anatomical structures. To our knowledge, there have been no published models that would include such a level of anatomical detail or that would be based on systematic segmentation supervised by experienced radiologist.

Our segmentation process has an obvious limitation, in that, the construction of the model requires a considerable amount of expertise and time. This is to a large extent a consequence of the complexity of the finger’s anatomy. Based on our experience in this study, it will be difficult—at least in the near future—to refrain from manual segmentation. Another limitation arises from the fact that there were no quantitative parameters that could assess the quality of the segmentation process and, thereby, the model created. The main reason is that we have already created the gold-standard model based on the expertise of an experienced radiologist.

Each voxel in our model can be assigned specific physical properties, and we have illustrated such an approach using Monte Carlo simulations of light transport within the human finger. As shown elsewhere [17], the Monte Carlo methodology can be successfully used for the detection of the early onset of arthritis in the proximal interphalangeal joint. Other possible extensions of the model include incorporating the elasto-mechanical properties of the tissues in the model, which would enable modeling of the finger joint’s mechanics. The simulation of heat transfer and the wave equation in an index finger is under way in our group, and to that end, we reformulated the model in terms of the finite-element methodology. Simulations of human finger surgeries would be another possible application. Finally, the model could be helpful in the optimization of the design of orthopedic devices and other instruments, such as pulse oximeters or devices for the detection of tissue chromophores.

A limitation of this study is that high-resolution MR images were available from only one male patient to build the finger model. The developed model, thus, does not cover the anatomic heterogeneity in a population. For example, Dolenec et al. [17] reported significant inter- and intra-subject variability in finger images using hyperspectral imaging. An obvious expansion of our study is building more finger models from high-resolution MR images of multiple female and male subjects to address anatomic heterogeneity. A larger number of finger models could also provide quantitative information about within-population variability, which could be in turn used for conducting sensitivity analysis.

**Figure 11:**
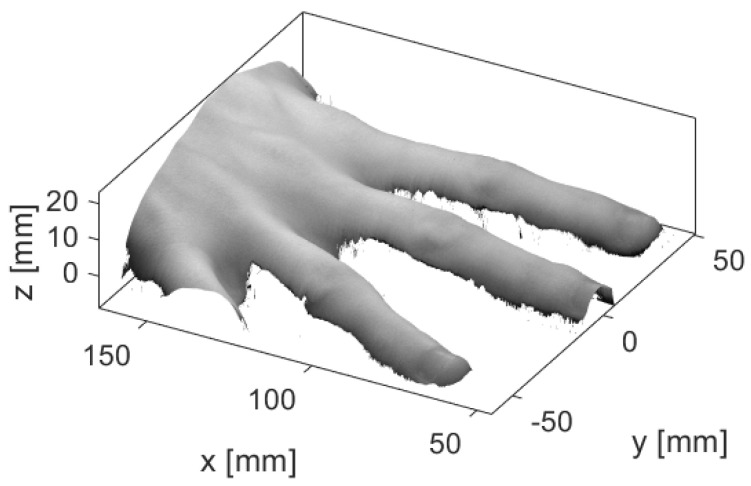
A 3D profile of a healthy human hand recorded using laser profilometry [19]. A spectral image recorded at 770 nm is overlaid over the 3D profile.

Another possibility is to fit the constructed finger model to the surface meshes obtained by optical profilometry imaging, i.e., a non-contact and non-invasive imaging technique for measuring the surface shapes of objects [29]. When using this modality, selected patterns are projected on the surface, and based on the pattern deformation, the surface shape is determined. An example of a surface mesh obtained during our pilot study in which surfaces of fingers were imaged is presented in Figure 11; a laser profilometry system, described in detail in [30], was used for imaging purposes. The image clearly shows the detailed surface shape of different fingers.

The finger model could be adapted to the surface meshes of individual fingers of subjects by using affine transformation of the 3D model [31]. The resulting transformed finger model would not necessarily correspond to the actual internal anatomy of the imaged finger since the surface data would not include this information. However, the external shape of the finger would agree with the shape of the imaged finger, e.g., its length, width, location of joints, and would, thereby, be partially solving the problem of the population heterogeneity.

In the past, a few studies also reported using finger joint models to simulate light transmission through healthy and inflamed joints. Lighter et al. [32] used a simple 2D model of a joint with only four different tissue types, where they attempted to simulate the inflammation as a non-physiologic, circular scattering in the joint cavity. Although simulations resulted in the transmitted light linear profiles roughly resembling their experimental findings, the profiles were featureless, not showing fine joint details. In addition, as it was a 2D model, it was not possible to simulate planar distributions of the transmitted light intensity. In the study by Milanic et al. [18], a 3D finger joint model was constructed from geometric shapes and 11 different tissues; the finger geometry was cylindrical with a spherical protrusion in the case of an inflamed joint. This model was capable of producing a planar distribution of transmitted light; however, due to the approximate geometry of the joint, the simulated distributions only roughly agreed with the experimental transmittance images. In comparison with the results of Lighter et al. [32] and Milanic et al. [18], simulations using our anatomically accurate model, shown in Figure 10, present an advancement as the simulated transmission images resemble the experimental images extremely well. Specifically, the distribution of lighter and darker areas in both the simulated and experimental images is highly similar and provides necessary details needed for the detection of arthritis. In addition, the simulated finger outline is also close to the experimental one, which is important when larger simulated datasets are used for training, e.g., a convolutional neural network (CNN) algorithm in case of inadequate experimental data.

## 5. Conclusions

In this study, high-resolution MR images along with careful segmentation proved useful in constructing an anatomically accurate model of the human index finger. As the model includes a number of tissue types, it can be particularly helpful in simulating various musculoskeletal disease processes in human joints.

## Figures and Tables

**Figure A1:**
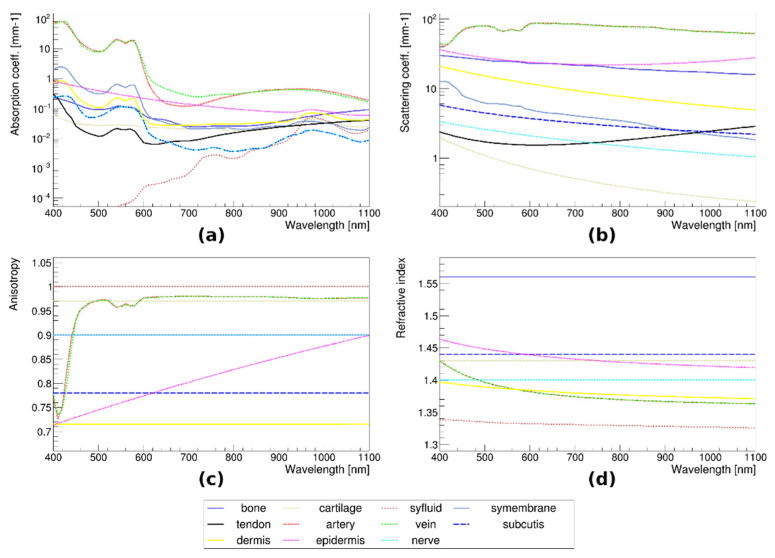
Wavelength dependence of the optical properties of unaffected tissues used in simulations: (**a**) absorption coefficient, (**b**) scattering coefficient, (**c**) anisotropy and (**d**) refractive index.

## Data Availability

The data that support the findings of this study are available from the corresponding author upon reasonable request.

## Appendix A

Figure A1 shows the absorption coefficient, scattering coefficient, anisotropy and refractive index for unaffected tissues used in simulations.
